# Inter-plot competition in hybrid maize multi-environment yield trials in Ethiopia can reduce rate of genetic gain

**DOI:** 10.1007/s00122-026-05255-0

**Published:** 2026-06-09

**Authors:** Tolera Keno, Emma Mace, Ian Godwin, David Jordan, Alison Kelly

**Affiliations:** 1 Queensland Alliance for Agriculture and Food Innovation, The University of Queensland, Warwick, Australia; 2 Ethiopian Institute of Agricultural Research, P.O. Box 2003, Addis Ababa, Ethiopia; 3 Queensland Alliance for Agriculture and Food Innovation, The University of Queensland, Brisbane, Australia

## Abstract

***Key messages*:**

The use of a competition model in the analysis of yield data from single-row hybrid maize METs improves the accuracy of genotype selection and thus the rate of genetic gain, while it ensures resource use efficiency in resource-constrained breeding programs.

**Abstract:**

Conducting large-scale multi-environment trials (METs) is practically challenging to breeding programs in Sub-Saharan Africa due to limitations of land, seed, and associated costs of phenotyping. As a result, early-generation hybrid maize trials are usually planted in single-row plots. In trials with single-row plots, genotypes compete with their neighbors for resources, and this biases the prediction of the genotype value. We demonstrate the impact of inter-plot competition in single-row multi-environment hybrid maize trials in Ethiopia through a motivating example assessing subsets of 478 maize hybrids in six trials in the 2019 and 2020 seasons. The trials were planted in partially replicated design with single-row plots. Field spatial variability and inter-plot competition were jointly modelled in a linear mixed model framework to partition the total genetic effect into direct and neighbour effects. Both direct and neighbour genetic effects differed across the trials confirming the presence of genotype by environment interaction (GEI) for these genetic components. There was a rank change in genotype performance between the selections from the competition model in comparison to the standard MET model. The proportion of mismatch in the top 20% of the selected genotypes from the competition model and the standard MET model ranged from 20–70% across the optimum nitrogen sites and up to 90% in the low nitrogen site. The results demonstrated that inter-plot competition biases grain yield predictions in single-row plot hybrid maize METs and thus reduces the rate of genetic gain, where the effect is particularly marked in low nitrogen environments.

## Introduction

In the early generation stages of hybrid improvement programs, many hybrids arising from test crosses are planted in the field every season. These large number of progeny are evaluated for yield and other traits of interest in a series of small-plot field trials grown across multiple locations, commonly referred to as multi-environment trials (METs). The Ethiopian mid-altitude maize breeding program develops maize hybrids for a target population of environments (TPE) that accounts for more than 50% of the maize production area in the country. This maize program generates 3000 to 6000 testcross hybrids every year and conducts phenotyping across two to four trial locations, within the TPE. The primary objective of these early generation trials is to select the hybrids with the best performance in order to maximize genetic gain moving forward in the breeding pipeline.

One of the practical challenges to maize breeding programs in Sub-Saharan Africa (SSA) is to plant, manage and evaluate large numbers of hybrids in multiple row plots in multiple locations. There is often limited seed and sometimes land available for hybrid testing and many trial operations are conducted manually and their costs scale with plot size. One of the solutions adopted in many SSA maize programs is the use of small unguarded single-row plots with few replicates in early generation METs. While this solution addresses the resource constraints of phenotyping, the impact of these very narrow plots should not be ignored in the analysis of MET data, as the hybrid selections from these small-plot field trials may not be predictive of hybrid performance when grown in a monoculture in farmers’ fields.

Genotypes grown in adjacent plots in field trials compete for available resources when little or no bordering is provided between plots (Kempton and Lockwood 1984; Rebetzke et al. 2014). This may mask the true worth of a genotype because variation in the competitive ability of genotypes in neighbouring plots may cause them to be perceived as better (or worse) than when competition is absent. There is evidence that indicates inter-plot competition affects grain yield in many crops, including maize (Castleberry 1986; David et al. 2001), soybean (Evans and Lewin 1986; Gedge et al. 1977), rice (Gomez 1972), sorghum (Hunt et al. 2013), wheat (Reynolds et al. 1994; Kramer et al. 1982; Foucteau et al. 2000), sugar beet (Kempton 1982), sugar cane (Stringer et al. 2011), field beans (Kempton and Lockwood 1984), and cotton (Moran‐Val and Miller, 1975). In many crops, differences in plant height (Austin and Blackwell 1980; May and Morrison 1986) or maturity (Genter 1958; Spitters 1980) have been reported to cause inter-plot competition, hence it was assumed that these traits are highly correlated with the aggressiveness of a genotype. Below-ground competition during progeny evaluations has also been known to be an important factor in inter-row competition effects in wheat (Donald 1963). In summary, many studies in different crops demonstrate that inter-plot competition seriously biases the assessment of genotype performance and thus can reduce the accuracy of the genetic prediction (Besag and Kempton 1986; Stringer and Cullis 2002; Stringer et al. 2011; Costa e Silva and Kerr 2013; Hunt et al. 2013).

The importance of inter-plot competition and its influence on maize grain yield in plant breeding trials has also been previously reported in the literature. Castleberry (1986) found that the grain yield of two-row plots of three hybrid trials decreased by approximately 30 kg ha^−1^ compared to when using self-guarded plots and concluded that the inter-plot competition was substantial and could not be ignored. Contrasting studies examined bordered and non-bordered plots in maize yield trials and found that competition effects were often statistically significant, but generally did not change the ranking of the hybrids for grain yield (Bowman 1989; Bänziger et al. 1995). However, these studies were conducted with very few genotypes. Additionally, the studies did not utilize the power of statistical models that account for field spatial variability to avoid its potential bias on the genetic value of the genotypes under evaluation.

In practice, inter-plot interference is often not considered in small-plot field trials due to the expected small impact across larger plot sizes comprised of multiple planting rows within each plot. Sometimes data collection and field operations are adjusted to minimize inter-plot interference. For example, the middle two rows of a plot may be harvested from four-row plots to minimise edge effects due to interference (Rebetzke et al. 2014). However, when plots sizes become small, the interference effects can be important, as is likely the case in single-row plots.

It is well-established that spatial variation impacts the accuracy of selection of genotypes from field trials and early statistical models focused on the similar performance of neighbouring plots (Wilkinson et al. 1983; Williams 1986; Gleeson and Cullis 1987). Statistical methods have also been developed to model interference between adjacent plots in field trials. The earliest model considered only treatment or genotypic interference (Pearce 1957), where the yield of each plot included the effect for the genotype grown on that plot, as well as the effect of genotypes grown in neighbouring plots. This model was reformulated as the producer-competitor model (Goldringer et al., 1994), by introducing terms for competitor effects from neighbouring plots. This treatment or genotypic model was extended to the phenotypic interference model by including terms for both random genotypic interference, as well as interference at the plot level, by modelling correlation between the residual errors (Besag and Kempton 1986). Stringer et al. (2011) formalized these models in a linear mixed model framework to include a random treatment interference model, and extended the residual variance structure to model both spatial variation and interference at the plot level. The extension to the residual variance model is through a constrained auto-regressive process, which includes positive correlation due to spatial variation, combined with negative correlation due to inter-plot interference. A comparison between the spatial LMM and a model including competition for random genetic effects as well as residual plot correlation has shown improvements in selection of genotypes based on individual trial results (Stringer et al. 2011).

While the linear mixed model has been extended to jointly account for spatial field heterogeneity and inter-plot competition in single field trials (Stringer et al. 2011), it has not been extended to model genotype by environment interaction (GEI) in MET data. A factor analysis (FA) variance structure has been proposed to capture the heterogeneity in genetic variance and genetic covariance that is common between trials in a MET (Piepho 1997; Smith et al. 2001). The form of this FA model is extended for the random treatment interference model to include both direct genotypic effects on each plot and neighbour genetic effects acting on adjacent plots across the narrowest plot dimension in the trial (Keno 2025).

A factor analytic MET model fitted to individual plot data from multiple field trials simultaneously models GEI using an FA variance structure for GEI effects, together with a spatial analysis model for each individual field trial (see Gilmour et al. 1997; Smith et al. 2001). This FA approach is extended to account for the effect of GEI in a competition MET (C-MET) model including both direct and neighbour effects, whilst simultaneously modelling inter-plot interference using a constrained autoregressive process for residual errors in each individual trial. A similar approach has been presented to account for the effect of GEI in forest trees (Belaber et al. 2021), adapting an individual tree model that is often used in forestry trials (Cappa et al. 2015; Tian et al. 2020). However, the model for inter-plot competition has not been developed or reported in a multi-environment study of field trials grown in a two-dimensional array of small plots, while accounting for GEI in direct and neighbour genetic effects across multiple environments.

In this paper, we formulate a competition multi-environment trial (C-MET) model to account for competition effects through both random genetic effects and residual plot errors in the presence of GEI. The impact of inter–plot competition in maize hybrid multi-environment trials grown in non-bordered single-row plots is explored to investigate whether inter-plot competition significantly affects genetic predictions of grain yield and the ranking of test hybrids in the Ethiopian maize MET data set.

## Materials and methods

### Plant material, trial design and trait measurement

The study was comprised of 478 maize hybrids generated from 106 maize inbred lines adapted to the mid-altitude sub-humid maize growing agro-ecology of Ethiopia. This agro-ecology represents the major maize growing areas which accounts for more than 50% of the maize production in the country. The hybrids were grown in six trials (sites) during the 2019 and 2020 main cropping season in Ethiopia. All trials were planted as a partially replicated (p–rep) design (Cullis et al. 2006), laid out as a rectangular array of plots indexed by columns and rows, with 8 columns and varying number of rows (Table 1). An experimental unit was a single row plot, 4.5 m long, spaced 0.75 m between rows and 0.25 m between plants. Two seeds were hand-planted per hill and subsequently hand-thinned to one plant per hill at 4 weeks after emergence, to give a final plant population density of 53,333 plants ha^–1^. The number of hybrids in each trial ranged from 240 to 259, and every trial had a different subset of hybrids, with differing concurrence sets of hybrids in common between each pair of trials (Table 2). The minimum number of hybrids in common between a pair of trials was 40.

**Table 1:** Number of columns, rows, genotypes in each trial, and altitude, latitude, and longitude of the sites where the trials are conducted

Site	Year	Management	Column	Row	Genotype	Altitude (m asl)	Latitude (^°^N)	Longitude (^°^E)
20BKO1	2020	Optimum N	8	56	240	1650	9.11	37.04
20BKO2	2020	Optimum N	8	58	250	1650	9.11	37.04
19BKON	2019	Optimum N	8	44	259	1650	9.11	37.04
19BKLN	2019	Low N	8	44	242	1610	9.09	37.04
20MLO1	2020	Optimum N	8	56	240	1550	8.39	39.32
20MLO2	2020	Optimum N	8	58	249	1550	8.39	39.32

**Table 2:** The number of genotypes tested at each of the six trials (diagonal), and the number of genotypes in common between each pair of trials (off-diagonal numbers)

Site	19BKLN	19BKON	20BKO1	20BKO2	20MLO1	20MLO2
19BKLN	242					
19BKON	242	259				
20BKO1	173	180	240			
20BKO2	40	40	91	250		
20MLO1	173	180	240	91	240	
20MLO2	40	40	91	249	91	249

At all the experimental sites, standard agronomic practices including weeding and appropriate fertilizer applications were followed. The ears were hand-harvested from each plot, then shelled using an ALMACO® maize plot sheller. The shelled grain per plot was weighed using a digital balance in kilograms per plot. The moisture content of the shelled grain (in percentage) was measured on a 200 g subsample of the grain using a Dickey–John® moisture meter. Grain yield (t ha^–1^) was calculated from the shelled grain weight adjusted to a standard grain moisture content of 12.5%.

### Statistical analysis

Consider a set of MET field trial data collected across *p* trials and *m* hybrids, where not all hybrids need to be included in every trial. Each trial is arranged as a two-dimensional array of *r*_*j*_ rows by *c*_*j*_ columns, for trial *j* = *1…p*, noting that trial dimensions can differ between trials within the MET set.

The linear mixed model for the MET trial data that accounts for spatial field variation, inter-plot competition and GEI is as follows:

1 $$\boldsymbol{y} = \boldsymbol{X\tau} + \boldsymbol{Z}_{c} \boldsymbol{u}_{c} + \boldsymbol{Z}_{o} \boldsymbol{u}_{o} + \boldsymbol{\varepsilon}$$

where ***y*** is an $n \times 1$ vector of data ordered as rows within columns within trials, τ is a vector of fixed effects (typically trial means), with associated design matrix, ***X***, ***u***_*o*_ is a vector of extraneous random effects (typically experimental design factors such as replicate, row and column), with design matrix ***Z***_*o*_, *ε* is an $n \times 1$ vector of residual errors. The genetic effects are denoted as $\boldsymbol{u}_{c} = \left( {\boldsymbol{u}_{d}^{\prime} ,\;\boldsymbol{u}_{n}^{\prime} } \right)$, where $\boldsymbol{u}_{c}$ is a $2mp \times 1$ vector, partitioned into the direct genetic effects and the neighbour genetic effects for each trial, and ***Z***_*c*_ = [***Z***_*g*_ ***N***_*g*_***Z***_*g*_], where ***Z***_*g*_ is the design matrix for hybrids, ***N***_*g*_ is an $n \times n$ first-order neighbour incidence matrix,

$${\boldsymbol{N}}_{g} = \oplus_{j = 1}^{p} {\boldsymbol{N}}_{{g_{j} }}$$ and $${\boldsymbol{N}}_{{g_{j} }} = {\boldsymbol{I}}_{{c_{j} }} \otimes {\boldsymbol{N}}_{{r_{j} }}$$.

where ${\boldsymbol{N}}_{{r_{j} }}$ is the neighbour incidence matrix in the row dimension, derived on a trial basis.

The variance–covariance matrix of the direct and neighbour genetic effects across trials is given by

$${\mathrm{var}}\left( {{\boldsymbol{u}}_{c} } \right) = {\boldsymbol{G}}_{c} \otimes {\boldsymbol{I}}_{m}$$

where, ${\boldsymbol{G}}_{c}$ has dimension $2p \times 2p.$ The form of this variance matrix in the competition MET model is

$${\boldsymbol{G}}_{c} = \left( {\begin{array}{*{20}c} {{\boldsymbol{G}}_{d} } & {\boldsymbol{G}_{dn}^\prime} \\ {{\boldsymbol{G}}_{dn} } & {{\boldsymbol{G}}_{n} } \\ \end{array} } \right)$$

where $${\boldsymbol{G}}_{d} = { } \left( {\begin{array}{*{20}c} {\sigma_{{d_{1} }}^{2} } & {} & {} \\ \vdots & \ddots \\ {\sigma_{{d_{1} d_{p} }} } & \cdots & {\sigma_{{d_{p} }}^{2} } \\ \end{array} } \right)$$, $${\boldsymbol{G}}_{n} = { } \left( {\begin{array}{*{20}c} {\sigma_{{n_{1} }}^{2} } & {} & {} \\ \vdots & \ddots \\ {\sigma_{{n_{1} n_{p} }} } & \cdots & {\sigma_{{n_{p} }}^{2} } \\ \end{array} } \right)$$,

and $${\boldsymbol{G}}_{dn} = { } \left( {\begin{array}{*{20}c} {\sigma_{{ d_{1} n_{1} }} } & \cdots & {\sigma_{{ d_{p} n_{1} }} } \\ \vdots & \ddots & \vdots \\ {\sigma_{ d_{1} {n_{p} }} } & \cdots & {\sigma_{{ d_{p} n_{p} }} } \\ \end{array} } \right)$$.

where $\sigma_{{d_{j} }}^{2}$ and $\sigma_{{n_{j} }}^{2}$ are the variances of the direct genetic effects and the neighbour genetic effects for trial *j*, respectively, $\sigma_{{d_{j} d_{j^{\prime}} }}$ and $\sigma_{{n_{j} n_{j^{\prime}} }}$ are the covariances of the direct genetic effects and the neighbour genetic effects between trials *j* and *j’*, respectively, and $\sigma_{{d_{j} n_{j^{\prime}} }}$ is the covariance between the direct and neighbour genetic effects, for trials *j* and *j’*.

The variance of the residual errors in the competition MET model is ${\mathrm{var}}\left( {\boldsymbol{\varepsilon}} \right) = \oplus_{j = 1}^{p} {\boldsymbol{R}}_{j}$, and ${\boldsymbol{R}}_{j} = \sigma_{j}^{2} \left( {{\boldsymbol{\varSigma}}_{{c_{j} }} \otimes{\boldsymbol{\varSigma}}_{{r_{j} }} } \right)$ is the residual variance matrix for trial *j,*

In the competition model, ${\boldsymbol{\varSigma}}_{{r_{j} }}$ can take the form of a constrained autoregressive process of order 2, with two correlation parameters, $\phi_{{r_{1} }}$ and $\phi_{{r_{2} }}$ for each trial, following the methodology of Stringer et al. (2011).

The LMM in Eq. (1) is denoted as the competition MET (C-MET) in this paper. Equation (1) simplifies to a standard MET, denoted S-MET in this paper, when ${\boldsymbol{u}}_{c} = {\boldsymbol{u}}_{d}$ where ${\boldsymbol{u}}_{d} \user2{ }$ is an $mp \times 1$ vector of genetic effects and ***Z***_*c*_ = ***Z***_*g*_. The residual model for an individual site simplifies to the standard spatial correlation model of an autoregressive process of order 1, with correlation parameter $\phi_{{r_{1} }}$ only, when $\phi_{{r_{2} }}$ = 0, following the methodology of Gilmour et al. (1997).

In practice, the sequence for fitting GEI variance models begins with a diagonal structure, modelling heterogeneous variance for direct and neighbour effects in individual trials and zero covariance, or independence, between these effects across pairs of trials.

The GEI effects can be formed using a factor analytic (FA) structure (Smith et al. 2001) for both direct and neighbour effects across trials. In the FA C-MET model, the vector of direct and neighbour GEI effects is ${\boldsymbol{u}}_{c} = \left( {{{\boldsymbol{\Lambda}}}_{c} \otimes {\boldsymbol{I}}_{m} } \right){\boldsymbol{f}} + {\boldsymbol{\delta}}_{c} \user2{ }$**,** where $${{\boldsymbol{\Lambda}}}_{c} = \user2{ }\left( {\begin{array}{*{20}c} {{{\boldsymbol{\Lambda}}}_{d} } \\ {{{\boldsymbol{\Lambda}}}_{n} } \\ \end{array} } \right)$$  and  $${\boldsymbol{\delta}}_{c} = \user2{ }\left( {\begin{array}{*{20}c} {{\boldsymbol{\delta}}_{d} } \\ {{\boldsymbol{\delta}}_{n} } \\ \end{array} } \right)$$.

In this FA regression model, ${{\boldsymbol{\Lambda}}}_{d}$ and ${{\boldsymbol{\Lambda}}}_{n} \user2{ }$ are $p \times k$ matrices of environmental loadings for the direct and neighbour effects, respectively for *k* factors, ***f*** is an $mk \times 1$ vector of hybrids scores common to both direct and neighbour effects and ${\boldsymbol{\delta}}_{d}$ and ${\boldsymbol{\delta}}_{n}$ are $mp \times 1$ vectors of residual GEI effects, respectively for the direct and neighbour effects. The vectors of random effects ***f***, ${\boldsymbol{\delta}}_{d}$ and ${\boldsymbol{\delta}}_{n}$ are assumed to be mutually independent and distributed as multivariate Gaussian with zero mean.

The joint distribution of ***f*** and ${\boldsymbol{\delta}}_{c} \user2{ }$ is given by:

$$\left( {\begin{array}{*{20}c} {\boldsymbol{f}} \\ {{\boldsymbol{\delta}}_{c} } \\ \end{array} } \right) \sim N\left[ {\left( {\begin{array}{*{20}c} \bf{0} \\ \bf{0} \\ \end{array} } \right), \left( {\begin{array}{*{20}c} {{\boldsymbol{I}}_{k} \otimes {\boldsymbol{I}}_{m} } & \bf{0} \\ \bf{0} & {{\boldsymbol{\varPsi}}_{c} \otimes {\boldsymbol{I}}_{m} } \\ \end{array} } \right)} \right]$$

where $${\boldsymbol{\varPsi}}_{c} = \user2{ }\left( {\begin{array}{*{20}c} {{\boldsymbol{\varPsi}}_{d} { }} & \bf{0} \\ \bf{0} & {{\boldsymbol{\varPsi}}_{n} } \\ \end{array} } \right)$$ ${\boldsymbol{\varPsi}}_{d}$ and ${\boldsymbol{\varPsi}}_{n}$ are a $p \times p$ diagonal matrix with elements $\psi_{{d_{j} }}$ and $\psi_{{n_{j} }}$ respectively for direct and neighbour effects in trial *j*, forming the heterogeneous specific variances in each trial (Smith et al. 2001). These variances can be interpreted as the residual or unexplained GEI variance for each environment.

In factor analytic form, the variance of the random GEI effects for the combined direct and neighbour effects, ${\boldsymbol{u}}_{c}$, is.

$$\boldsymbol{G}_{c} \otimes \boldsymbol{I}_{m} = \left( {\begin{array}{*{20}c} {\boldsymbol{G}_{d} } & {\boldsymbol{G}_{dn}^\prime} \\ {\boldsymbol{G}_{dn} } & {\boldsymbol{G}_{n} } \\ \end{array} } \right) \otimes \boldsymbol{I}_{m} ,$$

$$\boldsymbol{G}_{d} = \boldsymbol{\Lambda}_{d} \boldsymbol{\Lambda}_{d}^{\prime} + \boldsymbol{\Psi}_{d} ,\;\boldsymbol{G}_{n} = \boldsymbol{\Lambda}_{n} \boldsymbol{\Lambda}_{n}^{\prime} + \boldsymbol{\Psi}_{n} \,{\text {and}}\;\boldsymbol{G}_{dn} = \boldsymbol{\Lambda}_{n} \boldsymbol{\Lambda}_{d}^{\prime}$$

This results in a form of ***G***_*c*_ with heterogeneous variance for each environment and heterogenous covariance between each pair of environments, for both direct effects and neighbour effects, respectively. This form is an approximation to the unstructured form, and can be estimated when the variance matrix is of reduced rank (Thompson et al. 2003).

To interpret results from this model when selecting the best hybrids from the MET analysis, the genetic effects for the pure stand performance must be estimated. These pure stand performance values are the predicted values of genetic performance in a pure stand of that hybrid, in the absence of competition effects due to neighbouring plots (Goldringer et al. 1994). Pure stand effects for each hybrid are calculated as ***u***_*p* =_ ***u***_*d*_ + 2 ***u***_*n*_, and these genetic effects of pure stand performance are used to compare the competition model with the genetic effects from the standard MET model without competition. In practice, they are representative of the performance of the hybrids grown in a genetically identical monoculture in a farmer’s field.

All models were fitted in the R environment (R Core Team 2021, version 4.0.5) using the ASReml-R package, version 4.1 (Butler et al. 2017). The software estimates the variance parameters using residual maximum likelihood (REML), and forms empirical best linear unbiased estimates (BLUEs) of the fixed effects and empirical best linear unbiased predictions (BLUPs) of the random effects.

The code for the ASReml-R models invokes a general variance structure to partition direct and neighbour genetic effects across multiple trials and this involves complex ASReml-R syntax. The inclusion of a dummy variable in the factor analytic models is required to match the internal formulation of the conditional FA model within the ASReml-R estimation algorithm. Details of the R code are included in the supplementary material.

The sequential models fitted for S-MET and C-MET are compared using the Akaike Information Criterion (AIC) (Akaike 1998).

## Results

A total of four sequential models were fitted to the data using the standard LMM for GEI (S-MET) model and an additional five models were fitted to the data using the extension to the LMM for competition and GEI, denoted as the C-MET model. A summary of the models fitted is presented in Table 3. Overall, when comparing the sequential models fitted for S-MET and C-MET, the C-MET Model 8 had the minimum AIC value.

**Table 3:** Summary of the models fitted to the MET dataset. The first four sequential models are fitted to standard MET(Models 1 – 4) and the last five models were fitted to competition MET (C-MET)

Model	Structure	Variance model for Site:Genotype	Number of parameters	Log Likelihood	Akaike Information Criterion (AIC)	Computer time for model fitting (seconds)#
1	S-MET (Diag)	diag(Site): Genotype	31	−2266	4595	2
2	S-MET(FA1)	fa(Site,1): Genotype	37	−2163	4400	3
**3**	**S-MET(FA2)**	**fa(Site,2): Genotype**	**40**	−**2159**	**4397**	4
4	S-MET(FA3)	fa(Site,3): Genotype	43	−2158	4402	5
5	C-MET(Diag)	diag(Site):(direct + neighbour)	37	−2248	4569	4
6	C-MET(FA1)	fa(Site,1):(direct + neighbour)	49	−2113	4323	11
7	C-MET(FA2)	fa(Site,2):(direct + neighbour)	59	−2100	4318	28
**8**	**C-MET(FA3)**	**fa(Site,3):(direct + neighbour)**	**64**	−**2092**	**4312**	42
9	C-MET(FA4)	fa(Site,4):(direct + neighbour)	71	−2085	4313	34

^#^ Timing is measured in seconds on a laptop with the following specifications: 11th Gen Intel(R) Core(TM) i7-1800H @ 2.30GHz with 64GB RAM

A comparison of the C-MET model with the S-MET model for the analysis of individual trial data can be made between Models 1 and 5 in Table 3. The AIC value for the independent (diag) model for S-MET without competition (Model 1) was 4595 and the AIC was reduced to 4569 (Model 5) showing the improvement in model fit by including competition alone. This improvement was achieved by partitioning the total genetic value into direct and neighbour effects in each trial. This comparison ignores GEI and demonstrates the improvement in model fit due to partitioning for direct and neighbour effects at each trial, without the added complexity of modelling GEI.

Further improvements in model fit were achieved by modelling competition through direct and neighbour effects across environments (GEI). Modelling GEI in the S-MET model showed an improvement in model fit over the independence (diag) model (Model 1) for the S-MET model, with the best FA model being that of order 2 (Model 3). For the C-MET models with competition, Model 8 with an FA3 variance structure was the model of best fit, with an AIC value of 4312, showing significant improvement in model fit over the independence (diag) model with competition (Model 5).

The average grain yield (t/ha) across sites ranged from 2.88 at 19BKLN to 9.14 at 20BKO2 (Table 4), and estimates of variance parameters from the model of best fit for the S-MET FA2 and the C-MET FA3 models are also shown in Table 4. Genetic variance from the standard MET model ($\sigma_{g}^{2} )$ ranged from 0.056 at 19BKLN to 2.837 at 20BKO2. Considerable residual variance $\left( {\sigma_{\varepsilon }^{2} } \right)$ was observed with values ranging from 1.006 at 20BKO2 to 2.095 at 20MLO2. For the competition model, considerable genetic variance was observed for the genetic direct ($\sigma_{{g_{d} }}^{2} )$ and genetic neighbour ($\sigma_{{g_{n} }}^{2}$) components. The direct genetic variance across trials ranged from 0.029 at 19BKLN to 2.736 at 20BKO2. There was also substantial variation observed across locations for the genetic neighbour variance ranging from 0.102 at 20MLO1 to 0.143 at 20MLO2. In all trials except 19BKLN, the direct genetic variance was much greater than the neighbour genetic variance indicating direct effects were contributing more to the total genetic variation among the test hybrids at each site.

**Table 4:** Mean grain yield and REML estimates of variance components from the standard MET model (S-MET) and competition MET model (C-MET)

Site	Mean	S-MET		C-MET		
		$\sigma_{g}^{2}$	$\sigma_{\varepsilon }^{2}$	$\sigma_{{g_{d} }}^{2}$	$\sigma_{{g_{n} }}^{2}$	$\sigma_{{\varepsilon_{c} }}^{2}$
19BKLN	2.88	0.056	1.125	0.029	0.138	0.900
19BKON	8.69	2.173	1.03	2.261	0.135	0.880
20BKO1	8.98	1.957	1.541	2.261	0.104	1.197
20BKO2	9.14	2.837	1.006	2.736	0.120	0.759
20MLO1	8.12	1.168	1.379	1.065	0.102	1.252
20MLO2	7.82	1.784	2.095	1.416	0.143	1.931

The error variance from the C-MET FA3 model varied from 0.759 at 20BKO2 to 1.931 at 20MLO2. This model that accounts for the competition effect considerably reduced the error variances when compared to the model that ignores competition (S-MET FA2 model). The error variance at an individual site was reduced by 25% at 19BKLN, 17% at 19BKON, 29% at 20BKO, 33% at 20BKO2, 10% at 20MLO1 and 8.5% at 20MLO2. Furthermore, the competition variance (neighbour genetic variance) was less than the direct genetic variance at all sites except at 19BKLN. At this site, the competition variance was five times larger than the direct genetic variance. For the other five remaining sites the ratio of competition variance ($\sigma_{{g_{n} }}^{2}$) to direct genetic variance ($\sigma_{{g_{d} }}^{2}$) was 6% at 19BKON, 5% at 20BKO1, 4% at 20BKO2, 10% at 20MLO1 and 10% at 20MLO2 (Table 4).

**Fig. 1:**
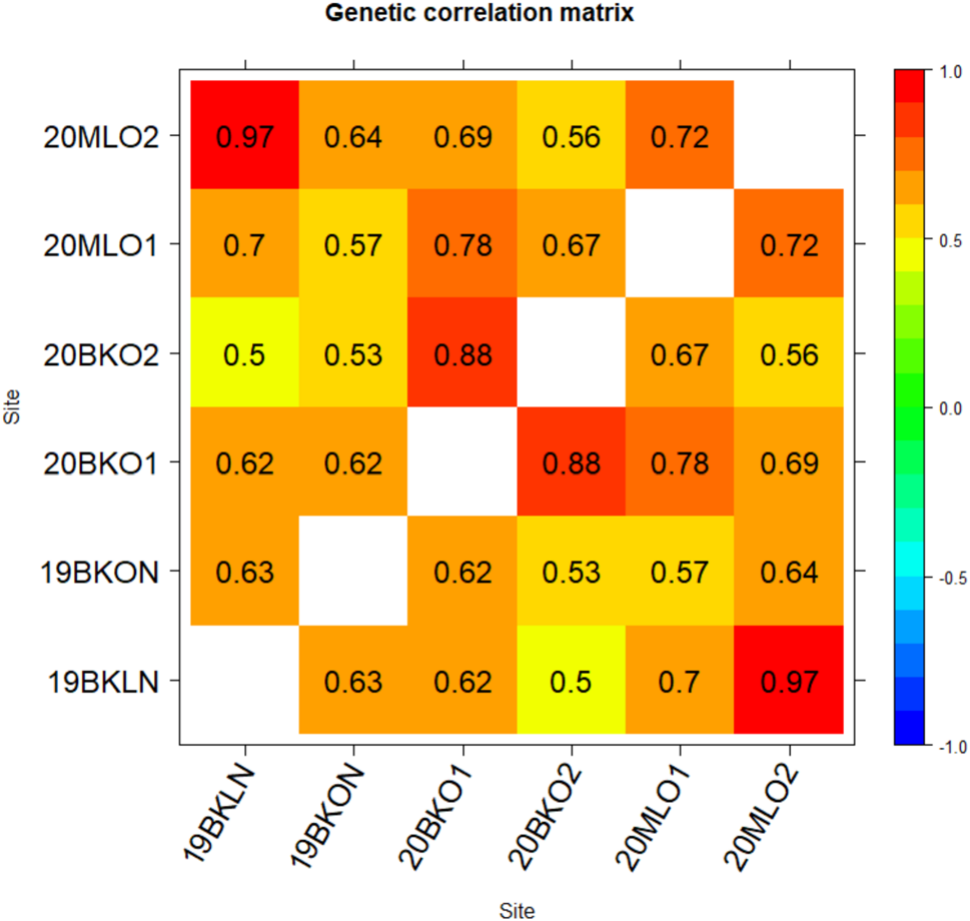
Genetic correlation between sites from the S-MET model for grain yield of maize hybrids tested at six sites in Ethiopia in 2019 and 2020

In order to better understand the magnitude and pattern of GEI, we compared the genetic correlation between trials from the S-MET FA2 model and C-MET FA3 model. Genetic correlations between trials from the S-MET model are presented in Fig. 1. From this model, genetic correlations between all pairs of trials were positive. Genetic correlations between the trials from the S-MET model ranged from 0.50 (19BKLN and 20BKO2) to 0.97 (19BKLN and 20MLO2) indicating moderate to minimal crossover GEI between sites. Simpler MET models assuming homogeneity of genetic variance and genetic covariance, fitting a compound symmetry model, were also considered, but the FA model was superior to GEI models with these simpler assumptions around GEI (results not presented).

**Fig. 2:**
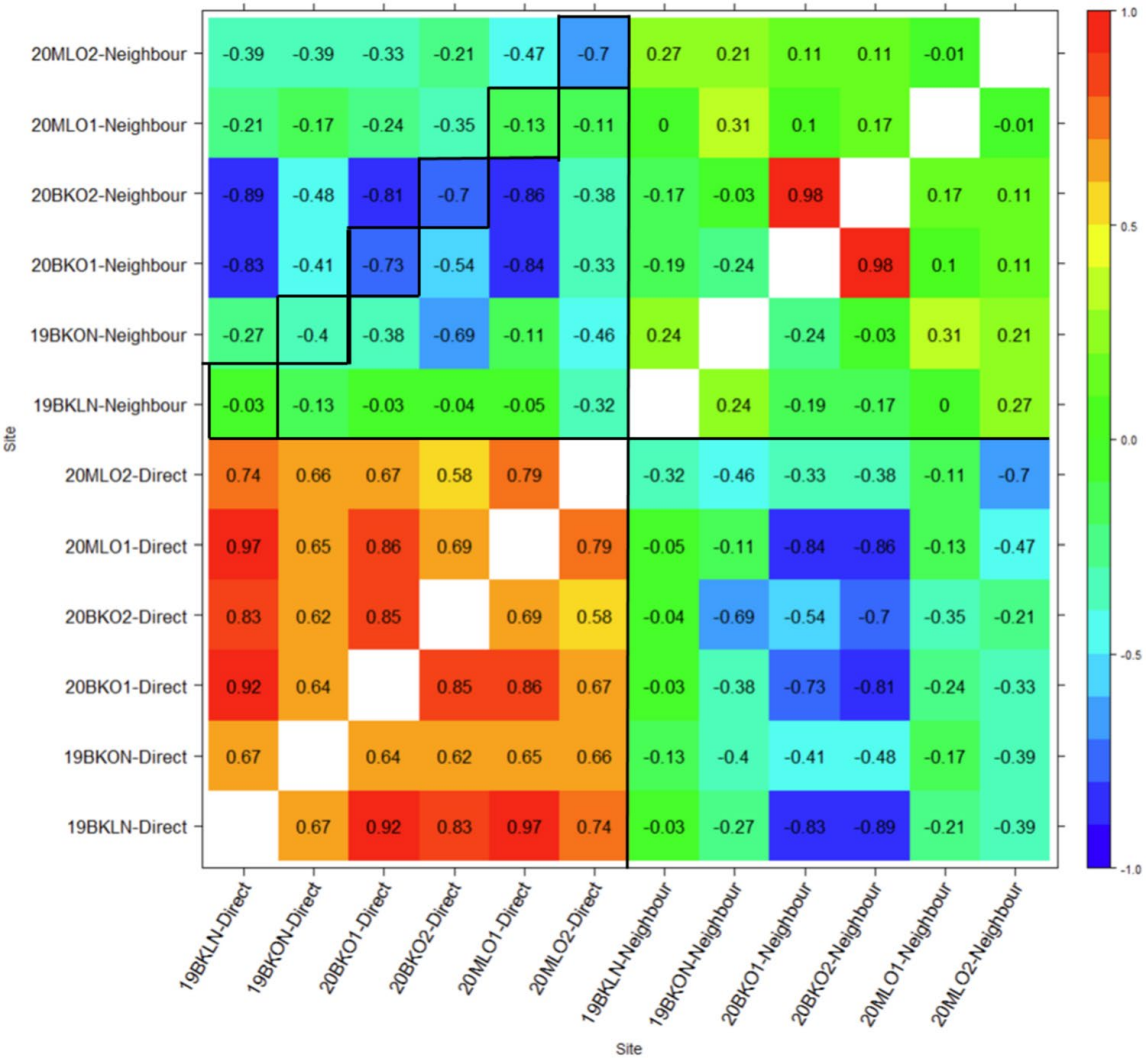
Genetic correlations between sites for direct genetic effects (lower left), neighbour genetic effects (upper right) and the correlation between direct and neighbour effects across sites (upper left or lower right). The diagonal values on the upper left show the relationship of neighbour and direct effects at the same site and the off-diagonal values shows the relationship of the effects at across sites

From the C-MET FA3 model, the direct genetic correlations ranged from 0.58 (20MLO2 and 20BKO2) to 0.97 (20MLO2 and 19BKLN) (Fig. 2, lower left). In this study, the pair of environments which had high genetic correlations from the S-MET model also exhibited high direct genetic correlations in the C-MET model. The direct genetic effects showed little change in the pattern of GEI that was observed in the S-MET model that ignores competition. However, there was a substantial reduction in the magnitude of GEI when neighbour effects are accounted for in the competition model. The average genetic correlation between sites from the S-MET model was 0.67, and this was increased to 0.74 when neighbour effects are accounted for in the C-MET model.

The neighbour genetic correlations between trials are shown in Fig. 2 (upper right). The neighbour genetic correlations between sites ranged from −0.24 (20BKO1 and 19BKON) to 0.98 (20BKO2 and 20BKO1). In this study, the neighbour genetic correlations between trials ranged from weak negative, through very low correlations to highly positive genetic correlations between sites, indicating that the neighbour genetic effects varied considerably across sites. These results indicate that there is strong GEI for competitive effect of hybrids across environments.

The genetic correlation values between direct genetic and neighbour genetic effects across sites from the C-MET FA3 model are presented in Fig. 2 (upper left or lower right). The values ranged from low negative to high negative, indicating the inverse relationship between direct and neighbour genetic effects across sites, as expected. That is, the hybrids which had large direct genetic effects had smaller neighbour genetic effects. Although there is substantial variation in genetic relationships between direct and neighbour genetic effects at the same site, ranging from −0.03 at 19BKLN to −0.73 at 20BKO1 (diagonal values), the values indicate a weak to strong inverse relationship at all the sites.

**Fig. 3:**
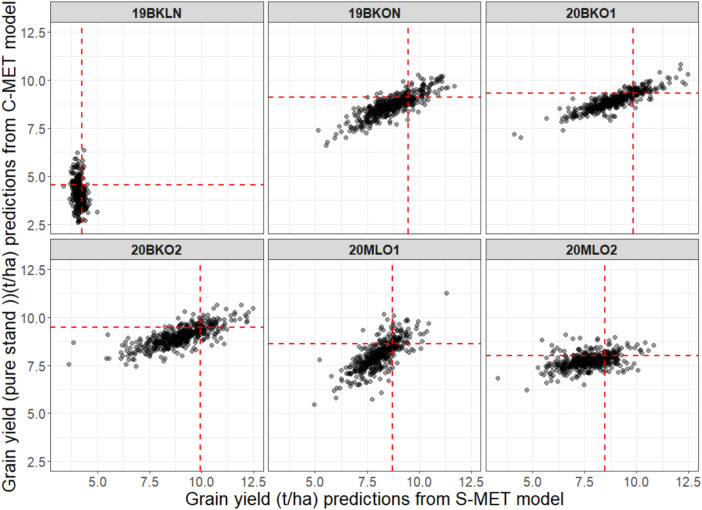
Pure stand predictions (BLUPs) from the C-MET model against the total genetic predictions from S-MET model. The dotted red lines indicate the top 20% selection of genotypes from the S-MET and C-MET models. The hybrids in the upper right quadrat are those hybrids in the top 20% that are common in both the standard and competition MET analysis results. The hybrids in the lower right quadrat of each plot are those in the top 20% of hybrids that are selected from a standard MET analysis, but would not be selected from the competition MET results. Conversely, the hybrids in the upper left quadrat are those hybrids selected in the top 20% of hybrids from the competition MET results that would not be selected from the standard MET analysis

The total genetic predictions from the model that accounts for competition are called “pure stand” effects (Goldringer et al. 1994). The scatter plot of the total genetic predictions from the S-MET FA2 model against the pure stand effects from the C-MET FA3 model at each trial are presented in Fig. 3. The genetic predictions between the S-MET model and the pure stand effects from the C-MET model showed substantial differences among hybrids between the two model predictions at each site. Consider a selection proportion of the top 20% of genotypes from the early generation testing to progress to the next stage in the breeding pipeline. The hybrids in the upper right quadrat of Fig. 3 are those hybrids in the top 20% that are common in both the S-MET and C-MET analysis results. The percentage of hybrids in the selected set in common between the two analysis methods range from 10 to 69% across sites. The hybrids in the lower right quadrat of each plot are those in the top 20% of hybrids that are selected from a standard MET analysis, but would not be selected from the competition MET results. Conversely, the hybrids in the upper left quadrat are those hybrids selected in the top 20% of hybrids from the competition MET results that would not be selected from the standard MET analysis.

**Fig. 4:**
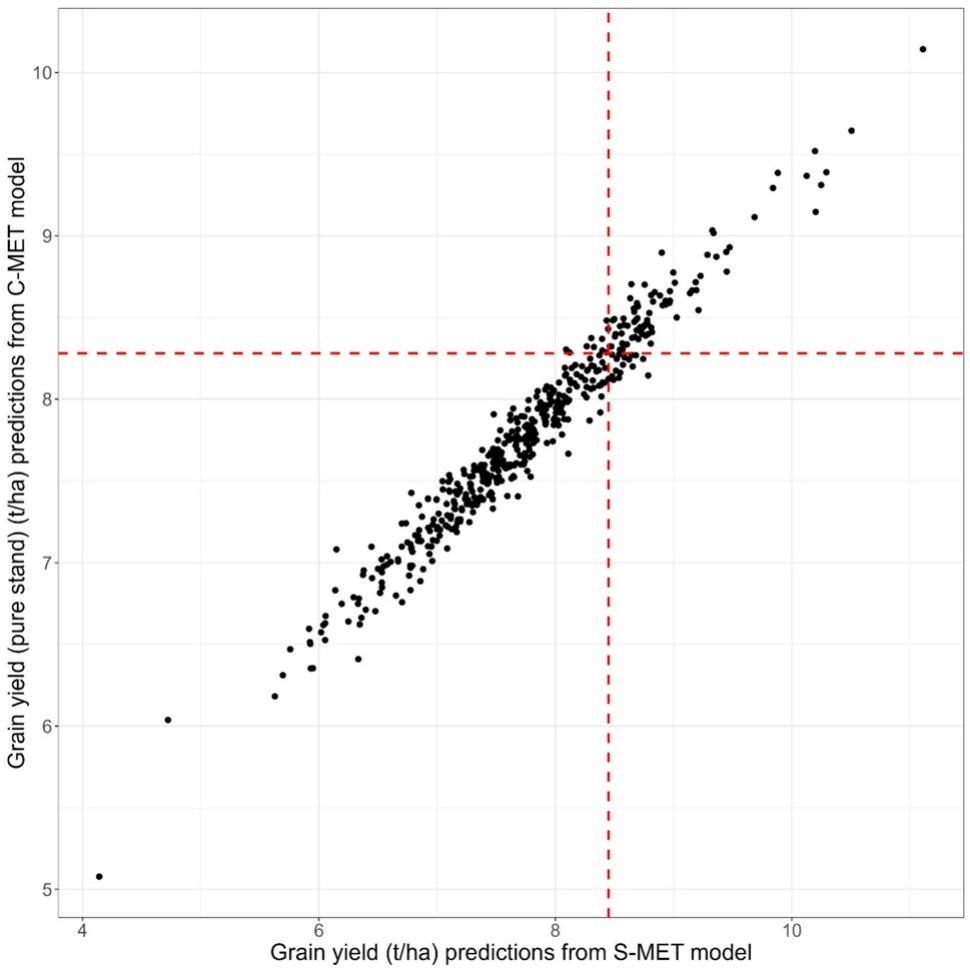
Overall genetic predictions averaged over six trials (19BKLN, 19BKON, 20BKO1, 20BKO2, 20MLO1, 20MLO2): Pure stand predictions (BLUPs) from the C-MET model against the genetic predictions from S-MET model. The vertical and horizontal dotted red lines indicate the top 20% selection of genotypes from the S-MET and C-MET models, respectively

The number of hybrids not in common (mismatched) in the top 20% of hybrids, or top 96 hybrids in number, between the S-MET and C-MET models differed from site-to-site. The proportion of mismatched hybrids in the top 20% of the hybrids between the two MET models was 40% for 19BKON, 31% for 20BKO1, 44% for 20MLO2, 35% for 20MLO1 and 33% for 20MLO2. More profound differences in hybrids ranking were observed in stressed sites, such as 19BKLN (90% mismatch). The mismatch proportions are reduced when considering the overall genetic response across trials. However, there are still 23% of hybrids mismatched for overall genetic predictions across the six trials between the S-MET and C-MET model (Fig. 4). These differences indicate that accounting for inter-plot competition in maize METs has a significant impact on selection of hybrids, thus affecting the genetic progress in the breeding program, even when overall genetic response is considered across multiple trials.

**Fig. 5:**
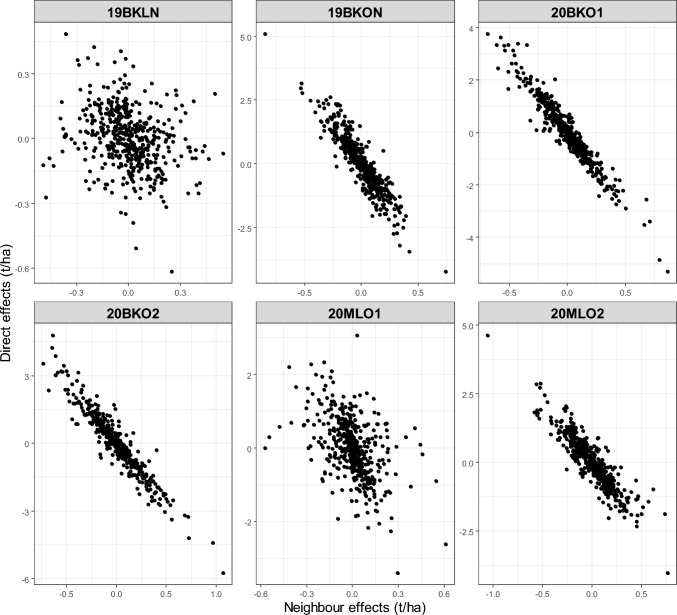
Scatter plot of the genetic predictions from neighbour plot effects against direct plot effects for maize hybrids tested at six sites in Ethiopia in 2019 and 2020

There is a strong underlying negative relationship between the direct and neighbour genetic predictions. This indicates that when hybrids do have high direct effects on the plot they were planted in, they exert a small effect on their neighbouring plots (Fig. 5). Therefore, the aggressive hybrids tend to have high direct genetic effects with a small yield impact on neighbouring plots, and the non-aggressive hybrids have low direct genetic effects, with a positive yield impact on neighbouring plots.

## Discussion

In the Ethiopian maize breeding program, and elsewhere in Africa, single row plots are widely used in early generation maize breeding yield trials due to limitations of land and resources for phenotyping. In these trials, inter-plot competition potentially biases grain yield predictions and this in turn reduces the rate of genetic gain in breeding programs, through sub-optimal selection decisions. In this study on single row maize trials, the approaches of (Stringer et al. 2011) have been extended to model the effect of inter-plot competition across multiple trials in maize. Competition in a MET setting has previously been reported for forestry trials (Belaber et al. 2021), but to our knowledge, this study is the first to extend the model for inter-plot competition to account for the effect of GEI in a two-dimensional spatial array common in field crop breeding trials. The competition MET (C-MET) model partitions the total genetic effects into direct and neighbour effects and estimates the interaction of these genetic effects across sites through the estimation of genetic variances and covariances in an LMM framework. This allows for prediction of the competition effect and subsequent derivation of the pure stand performance of hybrids for selection in the breeding program. Competition has been detected in small-plot field trials for various different crops, and statistical models have been proposed to partition the plot yield into a genetic effect that is measured directly on the plot, and a competition effect due to hybrids in neighbouring plots (Pearce 1957, Besag and Kempton 1986, Goldringer et al. 1994). Accounting for inter plot competition in individual trials has been demonstrated to improve the accuracy of genetic prediction in sugar cane (Stringer et al. 2011) and sorghum (Hunt et al. 2013). Using this same LMM framework to model competition in individual trial analysis, the comparisons in this study for single row maize trials showed that partitioning the total genetic value into direct and neighbour effects improves the model fit substantially. Comparing the independent site analysis from the S-MET (Model 1) and the C-MET model (Model 5) (Table 3), showed that the competition model had a lower AIC and higher REML loglikelihood, indicating it gave a better fit to the individual trial data. These findings indicate that inter-plot competition in single row plots in maize trials can be substantial, and these findings support the prior work of Castleberry (1986) who showed the same for two-row plots.

Extending the MET model using a FA regression structure for GEI effects across trials, further improves the model fit for both the S-MET model without competition (Model 1 versus Model 3) and for the C-MET model with competition (Model 5 versus Model 8). This improvement when modelling the GEI effects between trials with multi-row plots has been shown in numerous studies over the past decades, and the FA variance structure is now widely used for the analysis of MET data (see for example Smith et al 2001; Kelly et al 2007; Tolhurst et al. 2019). The improved accuracy of an FA model for GEI effects across trials is demonstrated in the maize data for both a standard MET approach and for the competition MET approach. Additionally, the FA model provided a significant improvement in model fit over those GEI models with simpler assumptions of homogeneous genetic variance and genetic covariance, such as a compound symmetry model, even when the correlations for this MET were all positive and of moderate (~ 0.5) to high (~ 0.8) magnitude.

The unique form of the FA model used for GEI effects in the C-MET model is a novel aspect of this study. The individual trial model partitions the genetic effects into direct and neighbour components (Stringer et al. 2011), and then the FA regression structure extends the genetic partitioning of direct and neighbour effects across environments. This partitioning adopts a structural form for the variance matrix that allows for correlation between the direct and neighbour effects, both within and between environments. The improvement in model fit between the best S-MET FA2 model, without competition, and the best C-MET FA3 model including both direct and neighbour effects shows a marked improvement in model fit. In addition to model improvement for an individual trial, as has been shown for this maize MET data as well as other studies, there are additional accuracy gains in modelling the covariance between these direct and neighbour effects across trials. In general, the results reported here showed that accounting for inter-plot competition in hybrid maize METs improved the model fit and reduced the residual variance compared with results from the S-MET model.

Both the S-MET and C-MET models proposed in this paper are fitted using the commercially available software package, ASReml-R in the R computing environment. While the models for competition effects are complex and require specific variance structures available in the ASReml-R package, the C-MET models were quick to fit and convergence of the REML loglikelihood was well-behaved. The C-MET models took slightly longer to fit than the S-MET models, but both models had good convergence properties for these MET data. The specific code for fitting the competition models is available in the supplementary material, but does rely on having access to the commercially available software package.

The information on the magnitude and pattern of GEI in breeding programs is very important for making selection decisions about the test hybrids and for devising future breeding strategies. Previous studies in maize in Ethiopia have highlighted the importance of GEI (Abakemal et al. 2016a, b; Wolde et al. 2019). However, these studies were typically conducted using single-row plot experiments, which could compromise the accuracy of the genetic information due to the inter-plot competition. Additionally, these studies did not account for spatial variability in the field which, together with competition, are major sources of bias in field experiments. In our study, a comparison of the genetic correlations between sites from the S-MET model and the direct genetic correlations from the C-MET model suggests that ignoring competition effects is likely to change the magnitude and pattern of estimates of GEI. This suggests that part of the genetic variance for competition in the improved model was attributed to GEI variance when competition was ignored, impacting hybrid selection at each site. This has wider implications in maize breeding in SSA, where maize-growing environments are very diverse, and GEI is common. While this is the first study to compare results for GEI between a standard MET analysis and a competition MET model in field crops, a similar trend has been reported in forest trees (Belaber et al. 2021).

The estimates of genetic correlations between sites for the neighbour effects varied, from very low to high, indicating the presence of high GEI for the competitive effect of the hybrids. Here it is important to note that the design of the experiment can impact on the estimation of these competition effects. Neighbour effects are estimated from a limited exposure of hybrids in one plot to the influence of very few other hybrids as neighbours. In the case of early generation breeding trials and under the resource constraints for phenotyping, this limited information is exacerbated by partial replication of hybrids within a trial, and the unbalanced nature of hybrid concurrence between trials. While it may not be optimal, the current study is representative of the trial composition and design in an early generation breeding study, and hence is appropriate for assessment of the impact of competition in this breeding context. If single-row plots are recommended, it will be important to understand the experimental design implications of neighbouring hybrids effects and both the partial balance of neighbour occurrence across the spatial field layout in a trial, as well as the impact of hybrid concurrence between trials in the MET set.

It is also important to note that competition between hybrids planted to neighbouring plots is intensified under stressed environments, as exemplified in the low N site in the current study, 19BKLN, where the competition variance was five times larger than the direct genetic variance. One qualification of this result, however, is that the estimate of genetic variance in the both the standard and competition MET model for 19BKLN was very low, relative to the other trials in this study. This can be expected for highly stressed sites, but it also impacts the accuracy of estimates for genetic effects at this trial. Other authors have observed that competition is intensified under low N stress in maize (Zhai et al. 2017), as the available nitrogen is exhausted by the aggressive hybrids and the sensitive hybrids are more disadvantaged. There are significant implications for hybrid selection in METs in such scenarios, as competition biases the true performance of hybrids and complicates selection. This suggests that there is a high level of uncertainty in predicting the genetic values of the hybrids planted in single-row plots with models that ignore competition effects. Additionally, the results indicated that this competition effect was more pronounced under stressed environments than optimum environments.

When crops are grown together is a monoculture of ‘like’ individuals, they are thought to redirect resources from producing competitive traits, for example increased height, to promoting reproductive growth (Rebetzke et al. 2014). A critical and practical question for breeding programs is whether there is a rank change between the predicted genetic values from a trial with single row plots ignoring competition and the pure stand predictions from the competition MET model. The proportion of mismatched hybrids in the top 20% of the predictions from S-MET and C-MET across environments indicated that there was a large selection discrepancy in ignoring the model that accounts for inter-plot competition in METs.

The proportion of mismatched hybrids was inversely proportional to the site mean, where the stressed sites had a larger proportion of mismatched hybrids in the top 20%, confirming that inter-plot competition is intensified under stressed sites. These stressed sites represent a significant proportion of the target population of maize production environments of small-scale farmers in sub-Saharan Africa (Falconnier et al. 2020). The results of this study suggest that any breeding program targeting these maize production environments selects against the performance of the hybrids if inter-plot completion is not accounted for. The impact of differences in the proportion of mismatched hybrids in the top 20% was reduced when considering average response across multiple environments. This finding will be impacted by the strength of the GEI and should be explored over a larger number and range of environmental conditions. Additionally, caution should be exercised when averaging across trials exhibiting strong GEI.

Previous studies examined bordered and non-bordered plots in maize yield trials and found that competition effects were often statistically significant, but generally did not change the ranking of the hybrids for grain yield (Bowman 1989; Bänziger et al. 1995). In our study, the re-ranking of hybrids performance between the predictions from S-MET and C-MET across sites indicated that ignoring inter-plot competition results in selection of hybrids whose performance is less than the observed. The statistical model that accounts for inter-plot competition can adjust for bias in these genetic estimates for yield potential, as a reproductive trait, by estimating the impact of competitive traits expressed in single-row plots. The limited comparison with other traits such as plant height and maturity explored in this study indicated that, not surprisingly, plant height has an impact on the competitive effects of hybrids. From the results of this study, it could be hypothesized that the mid-altitude maize program in Ethiopia is biasing selections for grain yield, in the absence of a competition model, to taller plants. However, this requires further study and justification.

## Conclusion

This study indicates that using a competition model in maize METs generally improves the accuracy of hybrid prediction and could be very useful for improving genetic gain in resource-limited breeding programs in SSA. The results from this study demonstrated that the yield prediction bias is greater in trials planted under stressed environments when the competition model is ignored. In Ethiopia, and in SSA in general, where maize is predominantly produced under a low input system by small-scale farmers (Shiferaw et al. 2011), inter–plot competition has greater negative impact on selections decisions in single row planted breeding trials unless accounted for with appropriate statistical models. The use of the competition MET model in hybrid selections targeting such representative production systems can increase the accuracy of selection and thus, the rate of genetic gain in breeding programs. Furthermore, the use of the competition MET model has broader application as it is feasible for breeding programs where resources are less limiting to shift from using two-row plots to single-row plot to double the number of test hybrids, for the same land resource, and use a statistical model to estimate inter-plot interference. This would ultimately contribute to improvement in the rate of genetic gain.

## Data Availability

The datasets generated during and/or analysed during the current study are available from the corresponding author on reasonable request.
